# Local Responses to a Global Pandemic: Women Mayors Lead the Way

**DOI:** 10.1017/S1743923X20000410

**Published:** 2020-07-14

**Authors:** Kendall D. Funk

**Affiliations:** Arizona State University

**Keywords:** COVID-19, local government, women's representation, policy priorities

## Abstract

Even before the novel coronavirus, COVID-19, was declared a pandemic, prominent women mayors in the United States enacted proactive and innovative policies to prevent local outbreaks and soften the social and economic repercussions. Several Black women mayors, in particular, have led the way in local pandemic response efforts. This article identifies four major features of these and other women mayors’ early responses. First, women mayors demonstrated proactive leadership even when faced with pushback. Second, these mayors advocated for transparent and evidence-based decision-making at all levels of government. Third, they enacted measures to protect vulnerable communities and reduce disparities. Fourth, they actively shared advice on best practices and lessons learned, and provided examples for other local leaders to follow. The article concludes by situating these responses in the larger research on gender and leadership and asks whether these women's actions are unique or part of a systematic trend of gendered responses to the pandemic.

When the World Health Organization declared COVID-19 a pandemic in March 2020, the U.S. federal government did little to stymie the virus's spread. Several subnational governments, however, responded quickly. Among those leading the way in response efforts are women mayors (Dittmar 2020). Several Black women mayors in particular, including San Francisco mayor London Breed, Chicago mayor Lori Lightfoot, and Atlanta mayor Keisha Lance Bottoms, have demonstrated remarkable leadership and ingenuity. These women mayors and others have not only provided leadership for their cities but also serve as role models for other leaders.

This article uses hypothesis-generating case studies (Levy 2008) of Democratic women mayors of large U.S. cities to theorize about potential systematic gendered responses to the pandemic. While these women may represent “extreme” cases (Seawright and Gerring 2008) given the underrepresentation of women, especially women of color, among U.S. mayors, the final section offers theoretical reasons why other women mayors may produce similar responses to the women featured here.

A focus on local governments is prudent as governors continue to reopen states and public health experts warn that local hotspots will emerge. Elected mayors and other local officials can implement policies to prevent local outbreaks and soften the pandemic's social and economic fallout. Examining mayors’ early responses to COVID-19 can provide an indication of how local leaders might—or should—respond in the future and whether mayors’ gender might affect their responses.

Four major features stand out among these women mayors’ responses. First, they demonstrated proactive leadership by quickly implementing preventive measures even as they received pushback from various stakeholders. Second, their decisions were transparent, evidence-based, and effectively communicated to the public. Third, they sought to not only prevent outbreaks but also protect vulnerable communities and reduce disparities. Fourth, they were generous in sharing best practices and lessons learned, providing models for others to emulate.

## PROACTIVE LEADERSHIP

In an interview with *Glamour*, San Francisco mayor Breed said that she realized the seriousness of COVID-19 in January (Kahn 2020). Following her realization, Breed acted quickly. She declared a state of emergency before a single case had been confirmed in San Francisco. She banned large gatherings in early March, effectively ending major sporting events. Shortly thereafter, she issued a citywide shelter-in-place order. Breed's early actions were criticized as overreaching and alarmist (Kahn 2020), but her swift actions undoubtedly prevented many deaths (Berman 2020).

Chicago mayor Lightfoot and Atlanta mayor Bottoms echoed Breed's impulse for action. In the same interview with *Glamour*, Lightfoot admonished, “I'm a Black woman in America. Nobody's coming to rescue me. I've got to get it for myself, and I feel the same way with what we need to do here in our city.” Bottoms urged, “We've got to be prepared for this regardless of what the federal preparations and the state preparations will be” (Kahn 2020).

These women mayors are not the only ones who responded proactively when federal and state responses seemed inadequate. Women mayors in Arizona, including Tucson's Regina Romero, Phoenix's Kate Gallego, and Flagstaff's Coral Evans, implemented preventive measures weeks before Arizona's governor issued a statewide stay-at-home order. These mayors then pushed back against Governor Doug Ducey's order for being too lax (Hassan 2020); Ducey responded with an executive order prohibiting local governments from closing businesses he deemed essential.

## TRANSPARENT AND EVIDENCE-BASED DECISIONS

Women mayors have also advocated for transparent, evidence-based decision-making. When Seattle became an early epicenter in the United States, Mayor Jenny Durkan was transparent with her constituents. She reasoned, “People are scared, confused, and getting mixed messages from the national and local level. I think people will trust their local leaders. You have to be transparent about the seriousness of the situation” (Bloomberg Harvard 2020). While the majority of the country was proceeding with business as usual, Durkan restricted large gatherings and engaged Seattle's research community to ramp up testing and epidemiological modeling (Durkan 2020).

In Arizona, when the statewide stay-at-home order was set to expire, Mayors Romero, Gallego, and Evans issued a statement urging the governor to follow all Centers for Disease Control and Prevention guidelines and to present clear data showing a decline in cases before reopening the state. Even more recently, Mayor LaToya Cantrell stated that New Orleans would not enter Phase 2 of reopening along with the rest of Louisiana. Cantrell explained her decision, stating, “We are watching the data, not the date. We don't yet have sufficient data to authorize opening up further at this point. All of our decisions must be grounded in science” (City of New Orleans Mayor's Office 2020).

## VULNERABLE COMMUNITIES

Women mayors have also expressed concerns over how the pandemic will affect vulnerable communities and exacerbate existing inequalities. Atlanta mayor Bottoms asked herself, “Who is going to be the most vulnerable? What will their needs be?” (Kahn 2020). These are questions that other women mayors seem to be asking—and answering—as well.

In a letter to congressional leaders, the African American Mayors Association warned that the pandemic will produce racial disparities (Owens 2020). This warning became a reality in Chicago, where Black residents make up a disproportionate share of COVID-19 cases and deaths (Wetli 2020). Mayor Lightfoot deemed these disparities “unacceptable” (Wetli 2020) and responded by adapting public transit, requiring health care providers to report demographic data with COVID-19 testing, and deploying a Racial Equity Rapid Response Team. In similar fashion, Tucson mayor Romero established the Somos Uno Resilience Fund to provide resources to members of the Latinx community regardless of their immigration status (Arce 2020).

Securing food and shelter for vulnerable communities and child care for essential workers was also integrated into their response plans. Breed and Durkan quickly issued moratoriums on evictions (Berman 2020; Durkan 2020). Breed increased shelter capacity to facilitate social distancing among San Francisco's homeless population (Berman 2020). Durkan, along with Bottoms and Cantrell, initiated programs to address food insecurity (Dittmar 2020; Kahn 2020). Durkan, Breed, and Lightfoot also launched child care programs for essential workers (Dittmar 2020; Kahn 2020).

## SHARING BEST PRACTICES

Early innovators, notably Durkan and Breed, have been generous in sharing their best practices and lessons learned. After managing Seattle's early outbreaks, Durkan wrote an op-ed for the *Washington Post* advising other mayors to act quickly, be transparent and honest, engage their research community, and anticipate outbreaks in vulnerable communities (Durkan 2020). Breed, meanwhile, shared her policies and resources with other cities, explaining, “As soon as someone asks, we send it. We're here to help" (Kahn 2020). Cities nationwide have replicated Breed's innovative policies, from her rigorous shelter-in-place order to the fee cap on delivery services. Atlanta mayor Bottoms confirms she watched Breed and reproduced Breed's stay-at-home order “almost word for word” (Kahn 2020).

Indeed, these women mayors have produced national models of effective pandemic responses. Among the first cities listed in the U.S. Conference of Mayors’ “City Guidelines and Best Practices” for COVID-19 are San Francisco, Seattle, Chicago, and Atlanta.^1^

## ARE PROACTIVE AND INNOVATIVE WOMEN MAYORS ANOMALIES?

Are these women anomalies or part of a systematic trend? While not all women mayors have demonstrated praiseworthy leadership—Las Vegas mayor Carolyn Goodman, for example, offered up her city as a “control group”—there are several reasons why other women mayors might act similarly to the ones highlighted here.^2^

Research finds that women tend to be more risk averse (Byrnes, Miller, and Schafer 1999) and supportive of government intervention (Schlesinger and Heldman 2001), and they prioritize social policy to a greater extent than men (Barnes, Beall, and Holman 2020; Funk and Philips 2019). If women hesitate to risk overrunning hospitals, prioritize health care over economic interests, and view government intervention favorably, then these characteristics might help explain women mayors’ proactive shutdown decisions.

There is also evidence that women mayors increase municipal transparency (Araujo and Tejedo-Romero 2018), and at least two reasons why they may support evidence-based health care policies. First, women politicians are more likely to have experience in the health care sector, and this can factor into their policy decisions (Barnes, Beall, and Holman 2020). Second, women are more likely to acknowledge limitations in their qualifications (Fox and Lawless 2011) and thus may be more likely to seek outside expertise to inform their decisions.

Women's greater emphasis on social issues (Barnes, Beall, and Holman 2020; Funk and Philips 2019) might also explain why these mayors’ response plans included social protections. And, insights from research on policy diffusion (Einstein, Glick, and Palmer 2019) and political role models (Stokes-Brown and Dolan 2010) suggests women mayors may look to one another for guidance. Thus, other women mayors may model their pandemic responses after these innovative leaders.

Finally, we must also acknowledge the role of intersectionality, especially the way partisanship and race factor into women mayors’ responses. Partisanship is major determinant of COVID-19 responses in the United States (Adolph et al. 2020), and the responses of non-Democratic women mayors have been notably less commendable than their Democratic counterparts. The women featured in this article are also trailblazers—often the first Black woman or lesbian woman to be elected mayor. The responses of Black women politicians, in particular, might be unique given their hypervisibility, progressiveness, and prioritization of “intersectionally marginalized populations” (Brown and Banks 2014). Thus, it is incumbent on future research to determine whether other women of color, White women, and conservative women leaders across contexts have also responded in proactive, transparent, evidence-based, and socially minded ways or whether these women mayors are truly anomalies.

## Footnotes

1. Available at https://www.usmayors.org/issues/covid-19/ (accessed July 3, 2020).

2. Carolyn Goodman, interview with Anderson Cooper on CNN, April 22, 2020, https://www.cnn.com/videos/politics/2020/04/22/las-vegas-control-group-coronavirus-mayor-goodman-vpx.cnn (accessed August 3, 2020).

## References

[ref1] Adolph, Christopher, Kenya Amano, Bree Bang-Jensen, Nancy Fullman, and John Wilkerson. 2020 “Pandemic Politics: Timing State-Level Social Distancing Responses to COVID-19.” medRxiv. 10.1101/2020.03.30.20046326. 32955556

[ref2] Araujo, Joaquim Filipe Ferraz Esteves, and Francisca Tejedo-Romero. 2018 “Does Gender Equality Affect Municipal Transparency: The Case of Spain.” Public Performance & Management Review 41 (1): 69–99.

[ref3] Arce, Javier. 2020 “Mayors of Tucson, Los Angeles Urge Federal Government for More Help for Latinos.” AZ Central, May 15. https://www.azcentral.com/story/news/local/arizona/2020/05/15/mayors-tucson-arizona-los-angeles-more-help-latinos-coronavirus/5202449002/ (accessed August 3, 2020).

[ref4] Barnes, Tiffany D., Victoria D. Beall, and Mirya R. Holman. 2020 “Pink-Collar Representation and Budgetary Outcomes in US States.” Legislative Studies Quarterly. Published online May 19. 10.1111/lsq.12286.

[ref5] Berman, Russell. 2020 “The City That Has Flattened the Coronavirus Curve.” *The Atlantic*, April 12. https://www.theatlantic.com/politics/archive/2020/04/coronavirus-san-francisco-london-breed/609808/ (accessed August 3, 2020).

[ref6] Bloomberg Harvard City Leadership Initiative. 2020 “Q&A with Seattle Mayor Jenny Durkan: On the Front Lines since January, Seattle Mayor Shares Her Take on Responding to an Outbreak.” March 31. https://www.cityleadership.harvard.edu/jenny-durkan-qa (accessed August 3, 2020).

[ref7] Brown, Nadia, and Kira Hudson Banks. 2014 “Black Women's Agenda Setting in the Maryland State Legislature.” Journal of African American Studies 18 (2): 164–80.

[ref8] Byrnes, James P., David C. Miller, and William D. Schafer. 1999 “Gender Differences in Risk Taking: A Meta-Analysis.” Psychological Bulletin 125 (3): 367–83.

[ref9] City of New Orleans Mayor's Office. 2020 “Orleans Parish Monitoring Data Closely, Will Remain under ‘Phase One’ Guidelines beyond June 5.” June 1. https://nola.gov/mayor/news/june-2020/orleans-parish-monitoring-data-closely,-will-remain-under-phase-one-guidelines-beyond-june-5/ (accessed August 3, 2020).

[ref10] Dittmar, Kelly. 2020 “Women on the Front Lines in Cities’ Fights against COVID-19.” Center for American Women and Politics, April 14. https://www.cawp.rutgers.edu/election-analysis/women-mayors-covid-19 (accessed August 3, 2020).

[ref11] Durkan, Jenny. 2020 “What Every Mayor Needs to Know about This Virus.” *Washington Post*, March 14. https://www.washingtonpost.com/opinions/2020/03/14/seattle-mayor-jenny-durkan-coronavirus/ (accessed August 3, 2020).

[ref12] Einstein, Katherine Levine, David M. Glick, and Maxwell Palmer. 2019 “City Learning: Evidence of Policy Information Diffusion from a Survey of US Mayors.” Political Research Quarterly 72 (1): 243–58.

[ref13] Fox, Richard L., and Jennifer L. Lawless. 2011 “Gendered Perceptions and Political Candidacies: A Central Barrier to Women's Equality in Electoral Politics.” American Journal of Political Science 55 (1): 59–73.

[ref14] Funk, Kendall D., and Andrew Q. Philips. 2019 “Representative Budgeting: Women Mayors and the Composition of Spending in Local Governments.” Political Research Quarterly 72 (1): 19–33.

[ref15] Hassan, Anita. 2020 “Arizona Mayors Slam COVID-19 Stay-at-Home Order That Allows Hair Salons, Golf Courses to Remain Open.” NBC News, April 1. https://www.nbcnews.com/news/us-news/arizona-mayors-slam-covid-19-stay-home-order-allows-hair-n1174186 (accessed August 3, 2020).

[ref16] Kahn, Mattie. 2020 “The Women Leading the Coronavirus Response from City Hall.” *Glamour*, May 4. https://www.glamour.com/story/women-mayors-leading-the-coronavirus-response (accessed August 3, 2020).

[ref17] Levy, Jack S. 2008 “Case Studies: Types, Designs, and Logics of Inference.” Conflict Management and Peace Science 25 (1): 1–18.

[ref18] Owens, Donna M. 2020 “Black Mayors Warn COVID-19 Could Hit Our Communities Especially Hard.” *Essence*, April 6. https://www.essence.com/feature/black-mayors-covid-19/ (accessed August 3, 2020).

[ref19] Schlesinger, Mark, and Caroline Heldman. 2001 “Gender Gap or Gender Gaps? New Perspectives on Support for Government Action and Policies.” Journal of Politics 63 (1): 59–92.

[ref20] Seawright, Jason, and John Gerring. 2008 “Case Selection Techniques in Case Study Research.” Political Research Quarterly 61 (2): 294–308.

[ref21] Stokes-Brown, Atiya Kai, and Kathleen Dolan. 2010 “Race, Gender, and Symbolic Representation: African American Female Candidates as Mobilizing Agents.” Journal of Elections, Public Opinion and Parties 20 (4): 473–94.

[ref22] Wetli, Patty. 2020 “Lightfoot Activates Rapid Response Team to Stem ‘Unacceptable’ Racial Disparity in COVID-19 Deaths.” WTTW News, April 6. https://news.wttw.com/2020/04/06/lightfoot-activates-rapid-response-team-stem-unacceptable-racial-disparity-covid-19 (accessed August 3, 2020).

